# Dominating Alzheimer's disease diagnosis with deep learning on sMRI and DTI-MD

**DOI:** 10.3389/fneur.2024.1444795

**Published:** 2024-08-15

**Authors:** Yuxia Li, Guanqun Chen, Guoxin Wang, Zhiyi Zhou, Shan An, Shipeng Dai, Yuxin Jin, Chao Zhang, Mingkai Zhang, Feng Yu

**Affiliations:** ^1^Department of Neurology, Tangshan Central Hospital, Hebei, China; ^2^Department of Neurology, Beijing Chao-Yang Hospital, Capital Medical University, Beijing, China; ^3^College of Biomedical Engineering and Instrument Science, Zhejiang University, Hangzhou, China; ^4^JD Health International Inc., Beijing, China; ^5^College of Science, Northeastern University, Shenyang, China; ^6^Department of Neurology, XuanWu Hospital of Capital Medical University, Beijing, China

**Keywords:** Alzheimer's disease, convolutional neural network, multi-modality, sMRI and DTI-MD, residual technique

## Abstract

**Background:**

Alzheimer's disease (AD) is a progressive and irreversible neurodegenerative disorder that has become one of the major health concerns for the elderly. Computer-aided AD diagnosis can assist doctors in quickly and accurately determining patients' severity and affected regions.

**Methods:**

In this paper, we propose a method called MADNet for computer-aided AD diagnosis using multimodal datasets. The method selects ResNet-10 as the backbone network, with dual-branch parallel extraction of discriminative features for AD classification. It incorporates long-range dependencies modeling using attention scores in the decision-making layer and fuses the features based on their importance across modalities. To validate the effectiveness of our proposed multimodal classification method, we construct a multimodal dataset based on the publicly available ADNI dataset and a collected XWNI dataset, which includes examples of AD, Mild Cognitive Impairment (MCI), and Cognitively Normal (CN).

**Results:**

On this dataset, we conduct binary classification experiments of AD vs. CN and MCI vs. CN, and demonstrate that our proposed method outperforms other traditional single-modal deep learning models. Furthermore, this conclusion also confirms the necessity of using multimodal sMRI and DTI data for computer-aided AD diagnosis, as these two modalities complement and convey information to each other. We visualize the feature maps extracted by MADNet using Grad-CAM, generating heatmaps that guide doctors' attention to important regions in patients' sMRI, which play a crucial role in the development of AD, establishing trust between human experts and machine learning models.

**Conclusion:**

We propose a simple yet effective multimodal deep convolutional neural network model MADNet that outperforms traditional deep learning methods that use a single-modality dataset for AD diagnosis.

## 1 Introduction

Alzheimer's disease (AD) is a progressive neurodegenerative disorder and one of the primary causes of cognitive decline and behavioral changes in the elderly (1–3). It significantly impairs patients' memory and cognition, leading to symptoms such as memory loss, disorientation, and difficulty understanding simple instructions, which profoundly impact daily life (4). Currently, there is no definitive cure for AD; thus, early diagnosis becomes particularly crucial for timely and effective medical intervention in individuals with cognitive impairments (5).

Traditionally, accurate diagnosis of AD has relied on doctors' extensive experience in analyzing a large amount of neuroimaging and clinical data to determine the symptoms (6). In recent years, computer-assisted disease diagnosis has gained increasing attention (7–10). For AD diagnosis, these methods primarily utilize computer vision techniques to extract discriminative features related to AD from neuroimaging, providing doctors with assisted diagnostic results. Specifically, structural magnetic resonance imaging (sMRI) measures structural changes in the brain, such as ventricular volume and cortical thickness (11). Functional magnetic resonance imaging (fMRI) investigates functional activity differences in patients during specific tasks by observing changes in brain oxygen levels (12). Positron emission tomography (PET) uses radioactive tracers to observe their distribution in the brain, providing insights into changes in neurotransmitters and metabolism in AD patients (13). Diffusion tensor imaging (DTI) analyzes the direction, extent, and integrity of neural fiber bundles by examining the diffusion process of water molecules in tissues (14).

Deep learning is a machine learning approach based on artificial neural networks that enables the extraction and recognition of nonlinear features through stacked neural networks (15). In recent years, deep learning techniques have achieved remarkable results in computer-aided disease diagnosis and have been widely applied in clinical practice (16, 17). HGGAN (18) generates multimodal brain network connectivity based on resting-state fMRI and DTI data, while MP-GAN (19) captures salient global features through a novel multidirectional mapping mechanism and efficiently visualizes the morphological features of AD by learning class-discriminative mappings for multiple classes with a single generator. Both hold potential application value for AD analysis. In medical image analysis, deep learning leverages large amounts of training data and high-performance computing platforms to learn and extract features from images (20). BSFL (21) decomposes the feature space into the union of the common and unique spaces for DTI and fMRI data through a decomposition-fusion framework, and then adaptively fuses them to analyze MCI. PALH (22) integrates prior-guided learning, adversarial learning, and hypergraph perception, capturing the complementarity within multimodal information through the fusion of learned representations, thereby improving the accuracy of disease diagnosis. Fuzzy-VGG (23) effectively enhances the accuracy of AD stage prediction based on brain MRI through fuzzy theory and a two-stage image enhancement strategy. MRL-AHF (24) enhances the accuracy of AD detection by extracting features through Graph Generative Adversarial Networks and Graph AutoEncoders, followed by the fusion of features from different modalities using an adversarial training strategy. However, existing deep learning models are often structurally complex, requiring a large amount of data, and may face gradient vanishing or model degradation issues. At the same time, the decision-making process of the models lacks interpretability, which can hinder doctors from understanding and trusting the diagnostic results of the models.

To address the aforementioned issues, we attempt to achieve high-precision AD diagnosis using sMRI and DTI. The choice of sMRI and DTI is primarily due to the fact that: sMRI reflects changes in brain structure, such as atrophy and lesions (25, 26). These local structural changes are associated with AD and can be effectively captured by convolutional neural networks. DTI measures the integrity and connectivity of neural fiber bundles. By analyzing DTI data, the degree of damage to white matter fiber bundles can be quantitatively evaluated. The combined use of sMRI and DTI can provide a more comprehensive perspective to assist in the diagnosis of AD. In this study, therefore, our main contributions are as follows:

(1) We propose a residual convolutional neural network model named MADNet for the classification of AD multimodal datasets. The residual networks within MADNet effectively tackle the issues of gradient vanishing and model degradation (27). MADNet is a straightforward yet potent network architecture that utilizes sMRI and DTI to accurately perform computer-aided diagnosis of AD.
(2) We construct a multimodal dataset based on the publicly available ADNI (Alzheimer's Disease Neuroimaging Initiative) dataset and the collected XWNI (Xuanwu Hospital Neuroimaging) dataset. This dataset includes 66 AD, 40 MCI, and 79 CN subjects, totaling 185 samples, which can be used to train and validate deep learning models for distinguishing these different groups.
(3) Our experimental results demonstrate that MADNet achieves superior performance, while visualization techniques reveal that MADNet focuses more on the cerebral cortex and ventricles, areas closely related to the development of AD, helping doctors establish trust in deep learning models.

## 2 Materials and methods

### 2.1 Dataset and preprocessing

The multimodal neuroimaging data used in this study are obtained from the AD Neuroimaging Initiative (ADNI, http://adni.loni.usc.edu) (28) and Xuanwu Hospital, Capital Medical University, Beijing. The modalities we utilize include sMRI and diffusion tensor imaging mean diffusivity (DTI-MD). The ADNI dataset is a large-scale collection of data that encompasses multiple neuroimaging modalities and has been widely utilized in AD research, including studies on disease progression, diagnosis, and treatment (29). In our research, we employ neuroimaging data from T1-weighted MRI and DTI modalities. The Xuanwu Hospital Neuroimaging (XWNI) dataset is obtained from Xuanwu Hospital, Capital Medical University, Beijing, China. This dataset includes data from AD, MCI, and CN patients utilizing sMRI, DTI, and PET modalities. Similarly, we utilize sMRI and DTI modalities for our investigation.

Due to the small sample size of the XWNI dataset, it is not suitable for use as a standalone training set and test set; therefore, we have combined the data from both datasets (XWNI and ADNI). For the AD vs. CN task, our training data consists of 764 CN samples and 121 AD samples. Specifically, we have 58 CN samples from XWNI and 706 CN samples from ADNI, along with 30 AD samples from XWNI and 91 AD samples from ADNI. In our testing data, we have 198 CN samples and 34 AD samples. Among these, 21 CN samples are from XWNI and 177 CN samples are from ADNI. Additionally, we have 11 AD samples from XWNI and 23 AD samples from ADNI. For the MCI vs. CN task, our training data includes 765 CN samples and 397 MCI samples. Out of these, we have 59 CN samples from XWNI and 706 CN samples from ADNI. Furthermore, we have 11 MCI samples from XWNI and 386 MCI samples from ADNI. In our testing data, we have 197 CN samples and 33 MCI samples. Specifically, 20 CN samples are from XWNI and 177 CN samples are from ADNI. Additionally, we have 10 MCI samples from XWNI and 23 MCI samples from ADNI.

### 2.2 Preprocessing

**Figure 1:**
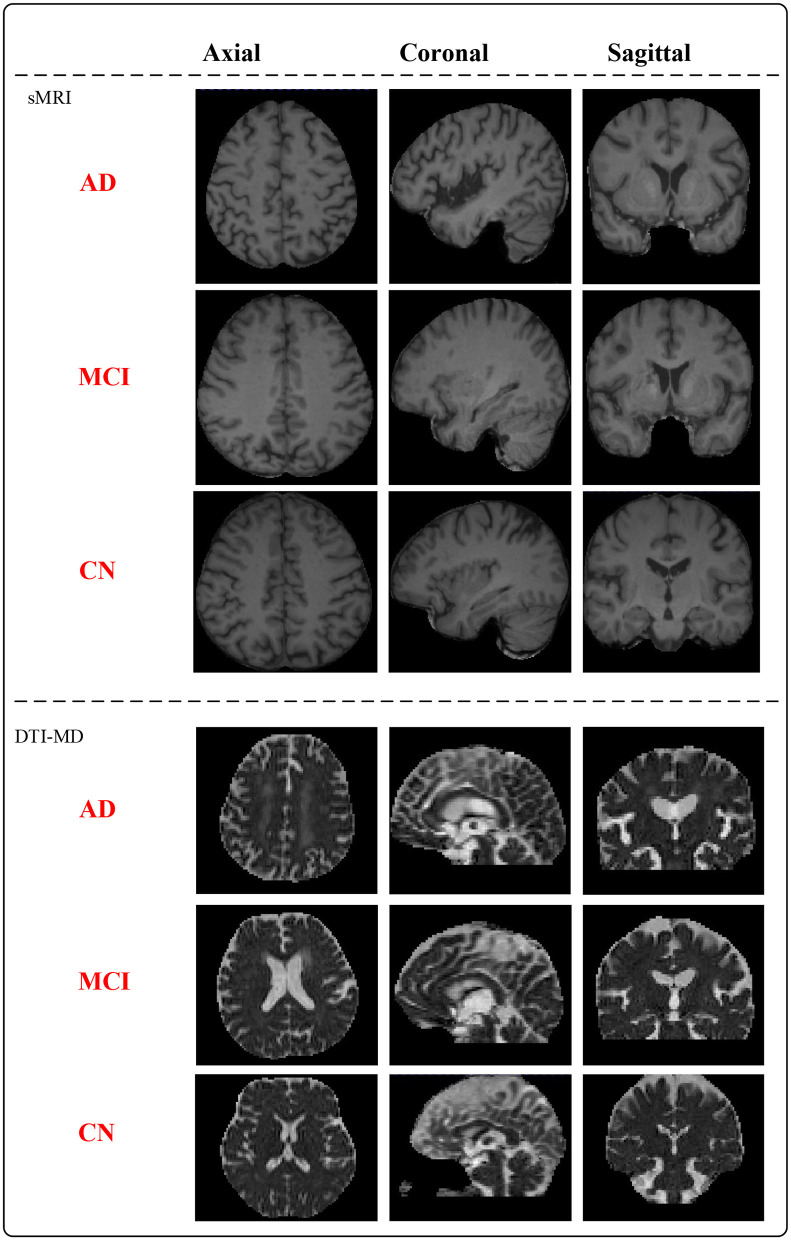
sMRI and DTI-MD brain structural images from the AD, MCI, and CN subjects.

MNI152_T1 is a standardized neuroimaging template developed collaboratively by McGill University, Montreal Neurological Institute (MNI), and the International Consortium for Brain Mapping (ICBM). This template is created based on the average brain morphology of a large number of participants and serves as a common reference space for researchers in neuroimaging data analysis. To ensure accurate brain region characterization, we have paired the raw T1-weighted sMRI with MNI152_T1_1mm considering the impact of spatial resolution on image quality (30). In the context of DTI in magnetic resonance imaging, mean diffusivity (MD) represents the average diffusion rate of water molecules and serves as a measure for describing their speed and direction within tissues (31). By utilizing DTI data, we calculate the average diffusion coefficients concerning different directions to obtain anisotropic mean diffusivity, enabling the assessment of the overall rate of water molecule diffusion in tissues. After preprocessing the data, the whole dataset (mix of XWNI and ADNI) consists of 66 AD subjects, 40 MCI subjects, and 79 CN subjects, totaling 185 patient samples. Figure 1 presents the preprocessed sMRI and DTI-MD modal images of the AD, MCI, and CN subjects. Both modalities provide three-dimensional (3D) data, from which deterministic images are extracted in the axial, coronal, and sagittal planes.

### 2.3 Single-modal feature extraction using residual convolutional neural network

**Figure 2:**
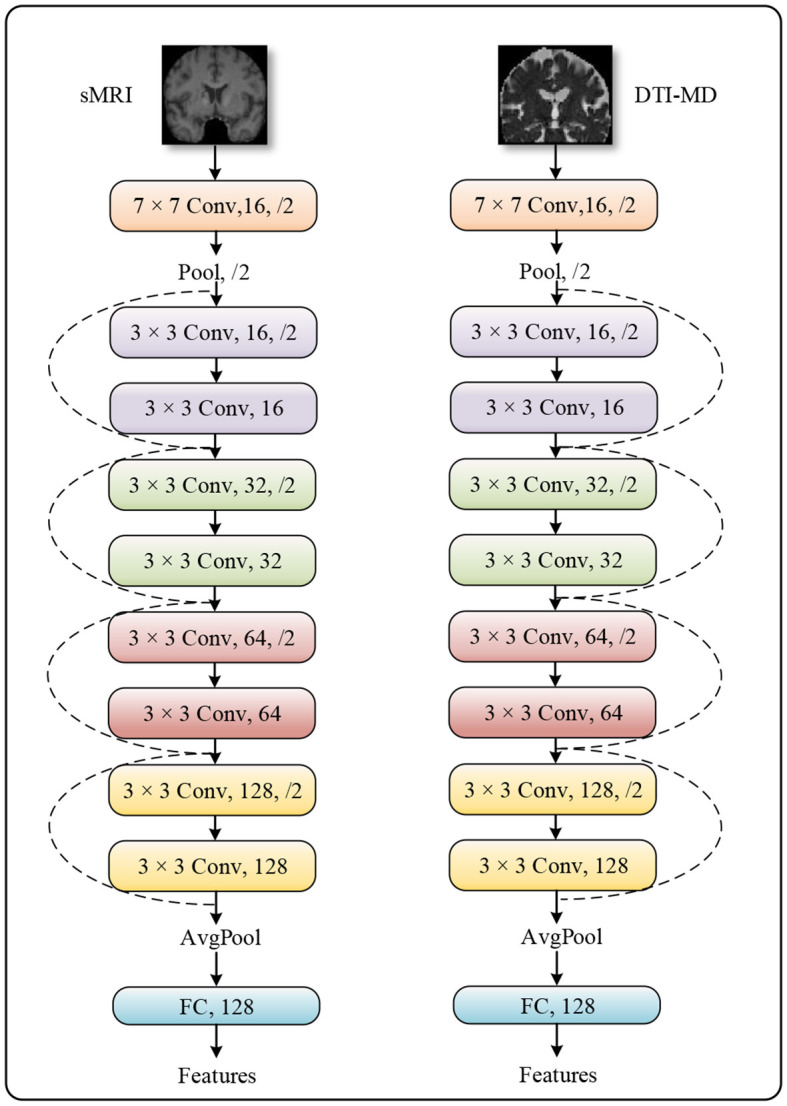
The specific structure of the feature extractor ResNet-10.

The method we propose follows a typical multimodal late fusion strategy, which necessitates accurately extracting discriminative features from the multimodal data in the early stages of the model, based on the fusion requirements (32, 33). We select ResNet-10 as the feature extractor, whose architecture is demonstrated in Figure 2. This is an artificial neural network model that combines convolutional operators with multiple residual branches. It is known for its ease of training and ability to capture local spatial features, making it well-suited for our task. The raw neural images are downscaled by ResNet-10 through five layers of 1/4 subsampling. After each subsampling, the channel dimension is doubled to compensate for the spatial information lost due to downsampling. After undergoing all convolutional operations in the network, the features are spatially aggregated into semantic information across channel dimensions by a global pooling layer.

### 2.4 Multi-modal joint construction of discriminative representations

In practice, the importance of different modalities of data for the same task may vary. Therefore, it is crucial to allow the model to adaptively adjust its perspective and allocate attention to each modality based on their respective importance when making decisions. In our method, We initially utilize ResNet-10 as the backbone network to extract discriminative features from two data modalities (27). This is because residual networks can resolve the vanishing gradient problem in deep network training through the introduction of residual learning. ResNet adds skip connections or shortcuts, allowing gradients to flow directly to shallower layers of the network, thereby enhancing the training efficiency and accuracy of the network. ResNet-10 is a variant of ResNet with fewer layers, making it effective even when computational resources are limited while maintaining the core advantages of residual networks. Secondly, ResNet-10 combines convolutional operators with multiple residual branches, making it highly suitable for automatic feature extraction in medical image analysis. Lastly, ResNet-10 downsizes the original neural images through five layers of 1/4 subsampling, and after each subsampling, the channel dimension is doubled to compensate for the spatial information lost due to downsampling. In this way, the network can capture local structural changes through convolutional operations, which are associated with Alzheimer's disease.

**Figure 3:**
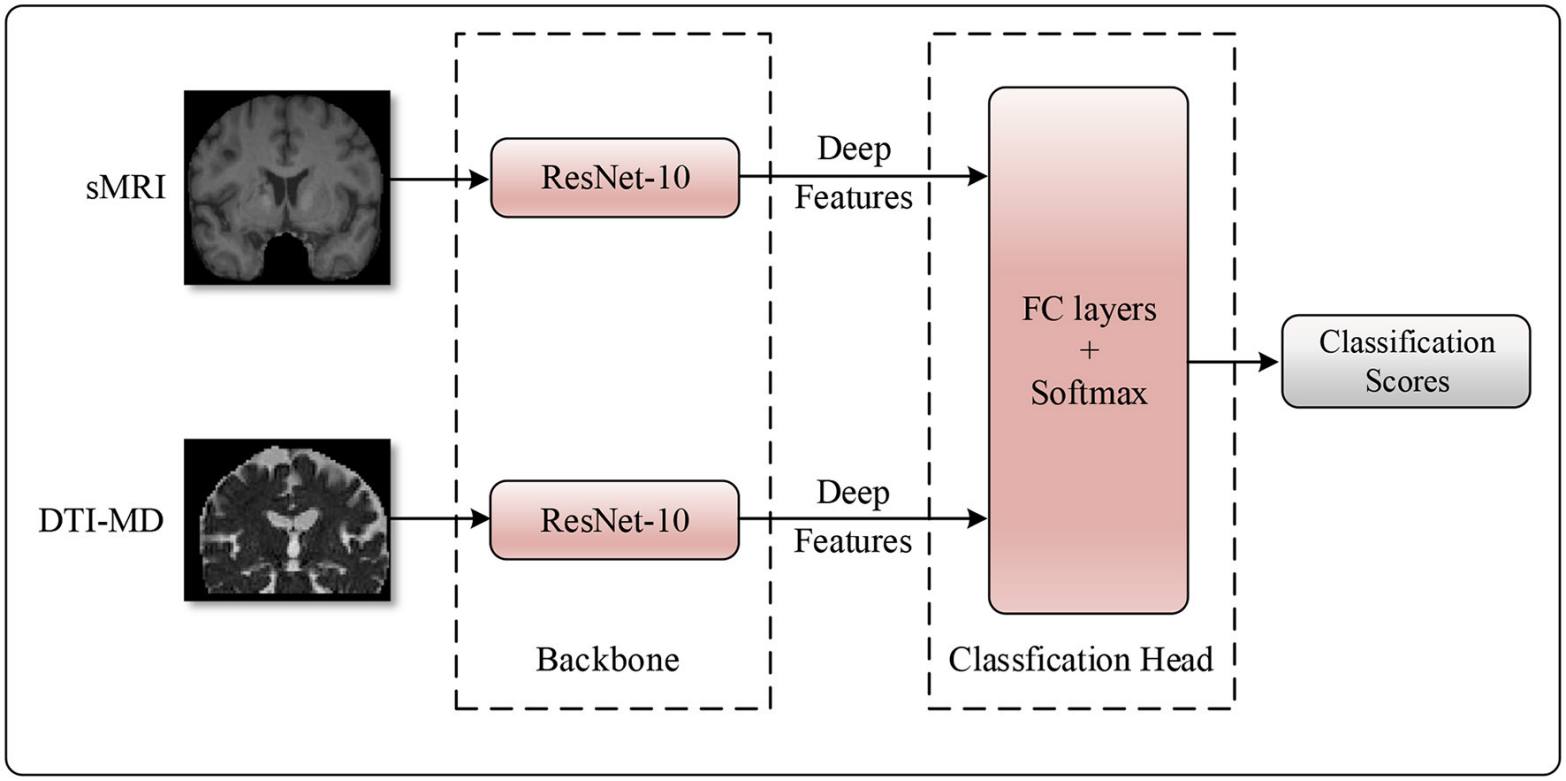
The structure of MADNet.

Next, taking into account the advantage of attention mechanisms in capturing long-range dependencies, we apply an attention mechanism at the decision-making layer to construct global representations between modalities (34). Specifically, the features from the two data modalities are concatenated along the channel dimension, followed by the use of a fully connected layer to obtain attention scores for each feature dimension. These attention scores are then element-wise multiplied with the multimodal features to obtain comprehensive features related to the importance of multiple data modalities. This approach allows the model to adaptively adjust its perspective and allocate attention based on the relative importance of each modality when making decisions. In this way, the model can more effectively integrate information from sMRI and DTI-MD, thereby providing more reliable evidence for the diagnosis of AD. The detailed structure of the model is illustrated in Figure 3.

## 3 Results

### 3.1 Implementation details

The proposed method in this study is implemented using Python 3.7.0 and PyTorch 1.10.0. We perform end-to-end training of the network using the AdamW optimizer, with an initial learning rate of 2e-4, which decays in a cosine annealing manner during iterations. To address the optimization challenges in the early stages of training, we employ a linear warm-up strategy, gradually increasing the learning rate for the first 30 epochs, with a total training duration of 200 epochs. The loss function utilizes cross-entropy loss, without any additional pretraining process. The batch size is set to 8, and the optimizer's weight decay is set to 5e-4. Considering the class imbalance in the dataset, we utilize weighted random sampling to balance the number of samples for each class. Specifically, we assign a weight to each class in the dataset that is inversely proportional to the number of its samples. During each training epoch, samples are randomly selected based on these weights to form a training batch, which means that samples from classes with higher weights are more likely to be included in the training batch. We train the model using this batch of weighted samples, ensuring that the model is not biased toward the majority classes but can better learn the characteristics of all classes. Additionally, to enhance data diversity, we apply horizontal flipping and random intensity transformations to the original medical images using the renowned medical image processing library, MONAI.

### 3.2 Comparison with other existing methods

Considering that there is currently no multimodal classification method for AD using both sMRI and DTI modalities, we compare it with existing single-modal AD diagnostic methods, performing classification separately on sMRI and DTI modal data to assess their performance. The compared methods included AMSNet (35), ResAttNet-10 (36), ResAttNet-18 (36), and Tencent YouTu's open-source pre-trained 3D medical image models, 3D-ResNet-10 and 3D-ResNet-18 (37). We evaluate the performance of these models based on accuracy (ACC), recall rate (REC), precision (PRE), F1 score, specificity (SPE), and AUC metrics.

### 3.3 Experimental results

We conduct binary classification experiments on AD vs. CN and MCI vs. CN respectively using multimodal datasets. Mathematically speaking, a higher numerical value for the six aforementioned metrics indicates better performance of the model. As shown in Tables 1, 2, our MADNet achieves better performance compared to existing single-modal classification methods in the AD vs. CN binary classification problem. This is attributed to the utilization of multi-modal data, which provides more reliable evidence for the diagnosis of AD using deep neural networks. Furthermore, we can observe that the 3D-ResNet-10 and 3D-ResNet-18 models perform better when using only sMRI modality data compared to using only DTI-MD modality data. However, the DA-MIDL, AMSNet, and ResAttNet models, which incorporate attention mechanisms, are more sensitive to the DTI-MD modality. This may be due to the attention mechanisms modeling global representations, which are more conducive to capturing discriminative features for AD diagnosis from the DTI-MD modality data.

**Table 1:** Quantitative comparison of our proposed MADNet and other existing methods only using sMRI for AD and CN binary classification.

**Method**	**ACC**	**REC**	**PRE**	**F1**	**SPE**	**AUC**
3D-ResNet-10	0.9394	0.7273	0.8276	0.7742	0.9747	0.9587
3D-ResNet-18	0.9524	0.7879	0.8667	0.8254	0.9798	0.9518
AMSNet	0.9264	0.6364	0.7222	0.7536	0.9495	**0.9637**
ResAttNet-10	0.9351	0.7576	0.7812	0.7692	0.9644	0.9247
ResAttNet-18	0.9307	0.6364	0.8400	0.7241	0.9798	0.9206
**MADNet (ours)**	**0.9654**	**0.8182**	**0.9310**	**0.8710**	**0.9899**	0.9597

The best results are in bold.

**Table 2:** Quantitative comparison of our proposed MADNet and other existing methods only using DTI-MD for AD and CN binary classification.

**Method**	**ACC**	**REC**	**PRE**	**F1**	**SPE**	**AUC**
3D-ResNet-10	0.9481	0.7273	0.8889	0.8000	0.9848	0.9242
3D-ResNet-18	0.9091	0.6970	0.6765	0.6866	0.9444	0.9276
AMSNet	0.9610	**0.8182**	0.9000	0.8571	0.9848	0.9343
ResAttNet-10	0.9524	0.7879	0.8667	0.8254	0.9798	0.9476
ResAttNet-18	0.9610	0.7879	0.9286	0.8525	**0.9899**	0.9343
**MADNet (ours)**	**0.9654**	**0.8182**	**0.9310**	**0.8710**	**0.9899**	**0.9597**

The best results are in bold.

MCI is a transitional stage from CN to AD. The diagnosis of MCI plays a crucial role in early intervention for AD patients. Tables 3, 4 present the performance of our proposed multimodal algorithm compared to existing methods in the MCI vs. CN binary classification task. It can be observed that MADNet achieves better performance in the MCI vs. CN binary classification task compared to existing methods that use single-modality approaches. MCI patients exhibit less pronounced changes in brain region structure compared to AD patients. As a result, the performance of MCI vs. CN binary classification is expected to be lower than that of AD vs. CN binary classification.

**Table 3:** Quantitative comparison of our proposed MADNet and other existing methods only using sMRI for MCI and CN binary classification.

**Method**	**ACC**	**REC**	**PRE**	**F1**	**SPE**	**AUC**
3D-ResNet-10	0.7767	0.6337	0.6809	0.6564	0.8492	0.8094
3D-ResNet-18	0.8200	0.8020	0.7043	0.7500	0.8291	0.8862
AMSNet	**0.8600**	**0.8182**	0.8172	0.7835	0.9146	0.8619
ResAttNet-10	0.7200	0.6337	0.5766	0.6038	0.7638	0.7533
ResAttNet-18	0.8567	0.8000	0.7959	**0.7839**	0.8995	**0.8999**
**MADNet (ours)**	0.8333	0.6337	**0.8312**	0.7191	**0.9347**	0.8634

The best results are in bold.

**Table 4:** Quantitative comparison of our proposed MADNet and other existing methods only using DTI-MD for MCI and CN binary classification.

**Method**	**ACC**	**REC**	**PRE**	**F1**	**SPE**	**AUC**
3D-ResNet-10	0.7933	**0.7030**	0.6893	0.6961	0.8392	**0.8666**
3D-ResNet-18	0.8000	0.6238	0.7412	0.6774	0.8894	0.7964
AMSNet	0.7967	0.6535	0.7174	0.6839	0.8693	0.8509
ResAttNet-10	0.7800	0.6436	0.6842	0.6633	0.8492	0.7905
ResAttNet-18	0.7933	**0.7030**	0.6893	0.6961	0.8392	**0.8666**
**MADNet (ours)**	**0.8333**	0.6337	**0.8312**	**0.7191**	**0.9347**	0.8634

The best results are in bold.

**Figure 4:**
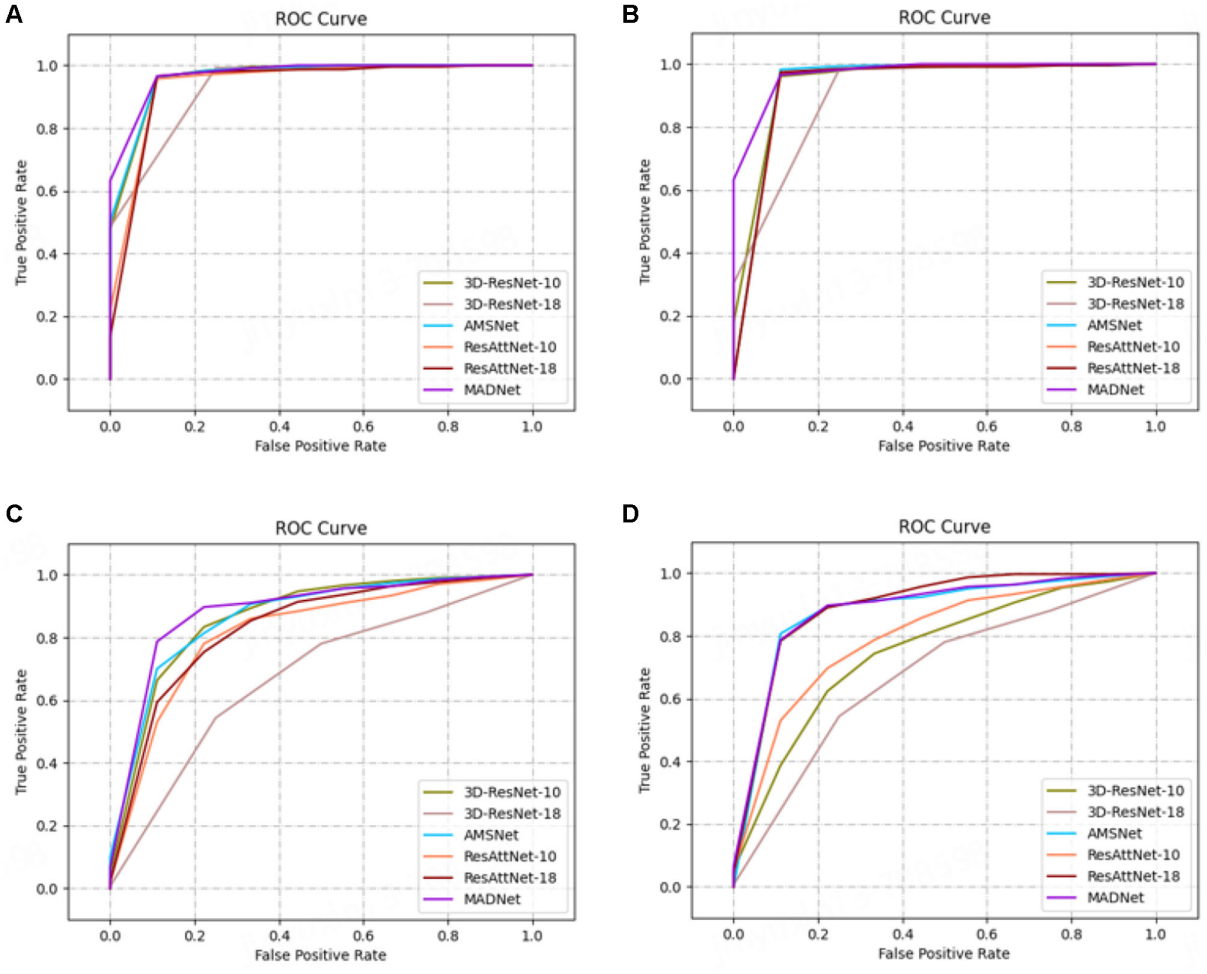
ROC curves. **(A, C)** are AD vs. CN task, and **(B, D)** are MCI vs. CN task. All methods except MADNet used sMRI data as inputs in **(A, C)**, and used DTI-MD data in **(B, D)**.

In our research, we employ receiver operating characteristic (ROC) analysis to evaluate the performance of different methods. Among the evaluated methods, our proposed approach demonstrates better performance, as evidenced by Figure 4.

### 3.4 Visualization

**Figure 5:**
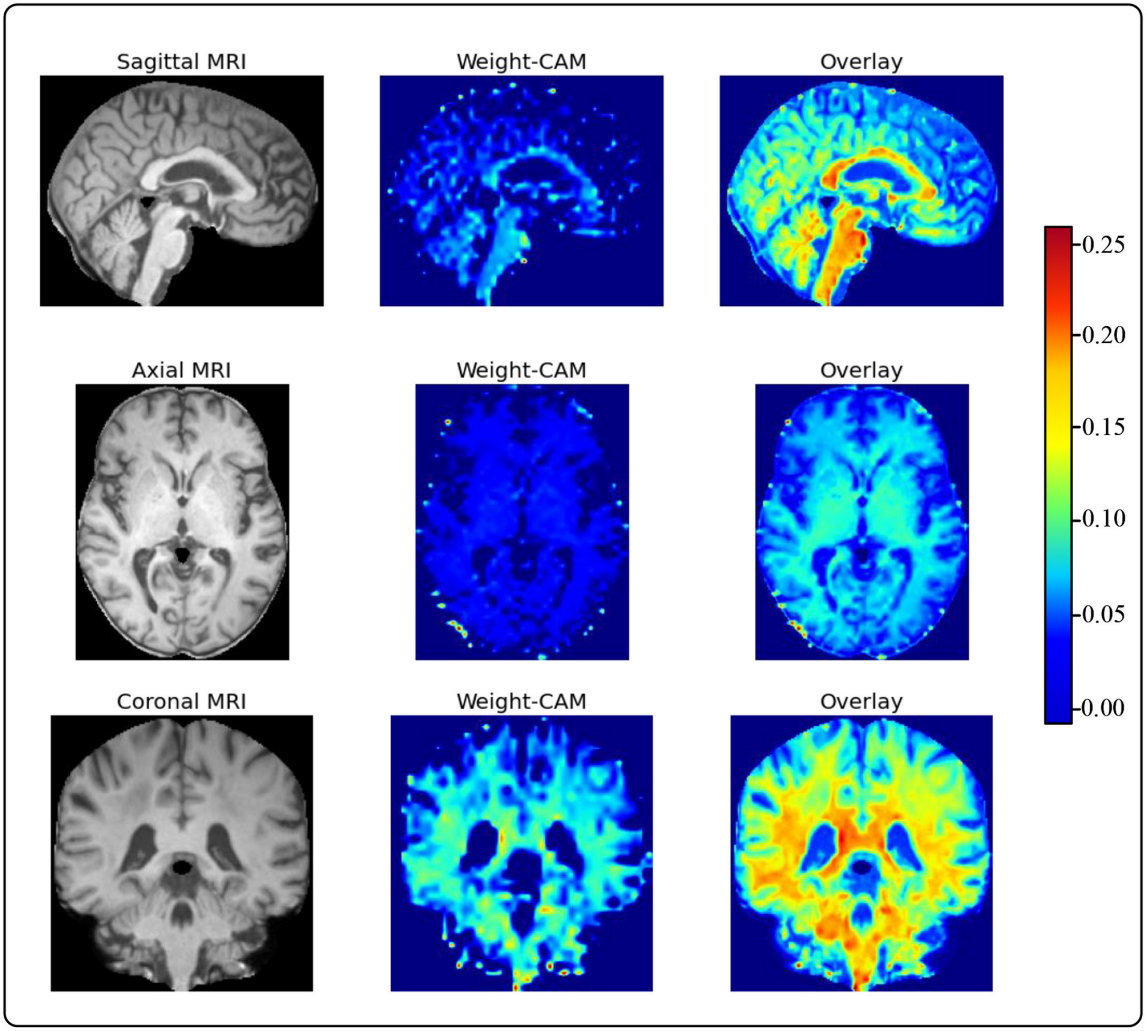
The visualized feature maps generated by Grad-CAM.

To provide accurate and reliable computer-aided diagnostic results for human experts, we use Grad-CAM to generate heatmaps of brain regions from sMRI slices. This is a model weight visualization technique that can help model developers identify the reasons behind the model's decisions. For instance, researchers utilize Grad-CAM to assess the model's capability to effectively recognize dark spots and flames within images (38). Furthermore, utilizing this technique in medical imaging can assist doctors in building trust with deep learning models. These heatmaps guide human doctors to focus on key areas of brain changes in AD patients, as shown in Figure 5 (39). We choose to visualize the feature map weights of a resolution of 36 × 44 × 36 and overlay them onto the raw sMRI. Through visualizing the heatmaps, we observe that our proposed model pays more attention to the cerebral cortex and ventricles. Upon consulting with the physicians in our team, we learn that the cerebral cortex and ventricles play crucial roles in AD. The cerebral cortex is the outer layer of the brain, responsible for processing complex cognitive functions such as memory, language, attention, and perception. Certain areas of the cerebral cortex in individuals with AD, particularly the hippocampus and entorhinal cortex, undergo significant atrophy and neuronal loss. This atrophy leads to a decline in cognitive functions and is one of the key indicators for the early diagnosis of AD. The ventricles are cavities within the brain, usually filled with cerebrospinal fluid. In patients with AD, the ventricles abnormally enlarge due to the shrinkage of brain tissue, leading to an increased spatial volume of the ventricular system. The enlargement of the ventricles can serve as a sign of AD progression and is associated with cognitive decline. This indicates that the features extracted by our model are not only meaningful for model decisions but also provide solid evidence for guiding human experts in quick and accurate lesion localization.

## 4 Discussions and conclusion

We propose MADNet, a model developed for computer-aided diagnosis of AD using sMRI and DTI-MD data, which has demonstrated superior performance over traditional single-modal deep learning methods. This multimodal approach emphasizes the complementarity of different neuroimaging datasets, offering a more nuanced understanding of the disease's progression and its impact on brain structure. Our findings indicate that the integration of sMRI and DTI-MD through a dual-branch parallel feature extraction enhances the model's ability to capture the intricate patterns associated with AD. The application of an attention mechanism at the decision-making layer allows for dynamic integration of multimodal features, considering the long-distance dependencies between modalities, which is crucial for accurate diagnosis. We utilize Grad-CAM for visualizing heatmaps to gain insights into the model's focus on the cerebral cortex and ventricles. These areas, known to be significantly affected in AD, further validate the model's capability to identify relevant pathological changes.

## 5 Limitation

This study presents a novel deep learning approach for AD diagnosis using sMRI and DTI-MD data through the MADNet. Despite the promising results, several limitations should be acknowledged to provide a comprehensive understanding of the scope and applicability of our findings:

(1) The current dataset, while multimodal, is limited in size, which may affect the generalizability of the model. Future studies should aim to include a larger and more diverse cohort to better represent the patient population and ensure the robustness of the model across different demographics.
(2) While sMRI and DTI-MD are utilized, the integration of additional modalities such as fMRI can provide temporal insights into brain structural changes associated with AD. Expanding the model to incorporate a broader range of imaging data can enhance diagnostic accuracy and provide a more holistic view of the disease progression.
(3) The MADNet is trained from scratch without the benefit of pretraining on large-scale datasets. Pretraining can potentially improve the model's ability to learn more complex features and representations, which could be particularly beneficial for medical imaging tasks where data can be scarce.

By addressing these limitations, we can develop artificial intelligence-assisted diagnostic models with higher precision in the future, ultimately contributing to the improvement of care and disease management for patients with AD and other conditions.

## Data availability statement

The original contributions presented in the study are included in the article/supplementary material, further inquiries can be directed to the corresponding authors.

## Ethics statement

The studies involving human participants were reviewed and approved by the Ethics Committee of Xuanwu Hospital Capital Medical University (Approval number: 2017046). Written informed consent to participate in this study was provided by the patients/participants or patients/participants' legal guardian/next of kin.

## Author contributions

YL: Conceptualization, Methodology, Project administration, Writing – review & editing, Data curation, Investigation. GC: Conceptualization, Methodology, Writing – review & editing, Project administration, Writing – original draft. GW: Conceptualization, Formal analysis, Supervision, Writing – review & editing. ZZ: Formal analysis, Methodology, Validation, Visualization, Writing – review & editing. SA: Project administration, Supervision, Writing – review & editing. SD: Investigation, Writing – review & editing. YJ: Validation, Visualization, Writing – review & editing. CZ: Investigation, Software, Writing – review & editing. MZ: Formal analysis, Resources, Writing – review & editing. FY: Supervision, Writing – review & editing.
